# Tetra­aqua­bis[3-(2-pyridylsulfan­yl)propionato *N*-oxide]nickel(II)

**DOI:** 10.1107/S1600536809011283

**Published:** 2009-03-31

**Authors:** B. Ravindran Durai Nayagam, Samuel Robinson Jebas, J. P. Edward Rajkumar, Dieter Schollmeyer

**Affiliations:** a Department of Chemistry, Popes College, Sawyerpuram 628 251, Tamilnadu, India; b Department of Physics, Karunya University, Karunya Nagar, Coimbatore 641 114, India; c Department of Physics, Popes College, Sawyerpuram 628 251, Tamilnadu, India; d Institut für Organische Chemie, Universität Mainz, Duesbergweg 10-14, 55099 Mainz, Germany

## Abstract

In the centrosymmetric title compound, [Ni(C_8_H_8_NO_3_S)_2_(H_2_O)_4_], the Ni^II^ ion, which lies on an inversion centre, is six coordinated by four water mol­ecules and two propionate O atoms from two 2-pyridylsulfanylpropionate N-oxide ligands, forming a slightly distorted octa­hedral geometry. An intra­molecular O—H⋯O hydrogen bond stabilizes the mol­ecular conformation. The crystal packing is consolidated by inter­molecular O—H⋯O and C—H⋯O hydrogen bonding.

## Related literature

For the biological activities of N-oxide derivatives, see: Bovin *et al.* (1992 ▶); Katsuyuki *et al.* (1991 ▶). Leonard *et al.* (1955 ▶); Lobana & Bhatia (1989 ▶); Symons & West (1985 ▶). For related literature, see: Jebas *et al.* (2005 ▶); Ravindran *et al.* (2008 ▶).
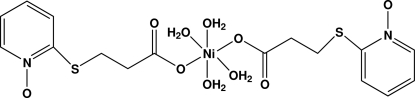


## Experimental

### 

#### Crystal data

[Ni(C_8_H_8_NO_3_S)_2_(H_2_O)_4_]
*M* *_r_* = 527.20
Triclinic, 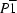

*a* = 4.8155 (5) Å
*b* = 8.7650 (10) Å
*c* = 12.9560 (15) Å
α = 86.400 (2)°
β = 79.501 (2)°
γ = 84.929 (2)°
*V* = 534.98 (10) Å^3^
*Z* = 1
Mo *K*α radiation
μ = 1.16 mm^−1^
*T* = 173 K
0.35 × 0.28 × 0.07 mm

#### Data collection

Bruker SMART APEXII CCD diffractometer
Absorption correction: multi-scan (*SADABS*; Sheldrick, 2008 ▶) *T*_min_ = 0.405, *T*_max_ = 0.492 (expected range = 0.759–0.922)
9627 measured reflections
2615 independent reflections
2501 reflections with *I* > 2σ(*I*)
*R*_int_ = 0.017

#### Refinement

*R*[*F* ^2^ > 2σ(*F* ^2^)] = 0.020
*wR*(*F* ^2^) = 0.055
*S* = 1.05
2615 reflections
142 parameters
H-atom parameters constrained
Δρ_max_ = 0.40 e Å^−3^
Δρ_min_ = −0.36 e Å^−3^

### 

Data collection: *APEX2* (Bruker, 2008 ▶); cell refinement: *APEX2*; data reduction: *APEX2*; program(s) used to solve structure: *SHELXS97* (Sheldrick, 2008 ▶); program(s) used to refine structure: *SHELXL97* (Sheldrick, 2008 ▶); molecular graphics: *SHELXTL* (Sheldrick, 2008 ▶); software used to prepare material for publication: *SHELXTL* and *PLATON* (Spek, 2009 ▶).

## Supplementary Material

Crystal structure: contains datablocks global, I. DOI: 10.1107/S1600536809011283/bt2915sup1.cif

Structure factors: contains datablocks I. DOI: 10.1107/S1600536809011283/bt2915Isup2.hkl

Additional supplementary materials:  crystallographic information; 3D view; checkCIF report

## Figures and Tables

**Table 1:** Selected bond lengths (Å)

Ni1—O13	2.0488 (8)
Ni1—O15	2.0644 (8)
Ni1—O14	2.0898 (8)
N1—O7	1.3154 (13)

**Table 2:** Hydrogen-bond geometry (Å, °)

*D*—H⋯*A*	*D*—H	H⋯*A*	*D*⋯*A*	*D*—H⋯*A*
O14—H14*A*⋯O12	0.81	1.84	2.6248 (12)	162
O14—H14*B*⋯O15^i^	0.81	2.27	2.9517 (12)	142
O14—H14*B*⋯O13^i^	0.81	2.64	3.2316 (12)	131
O15—H15*A*⋯O7^ii^	0.82	1.83	2.6469 (12)	172
O15—H15*B*⋯O13^iii^	0.83	1.83	2.6570 (11)	172
C4—H4⋯O12^iv^	0.95	2.48	3.2044 (17)	133
C6—H6⋯O14^v^	0.95	2.46	3.2515 (16)	140
C10—H10*B*⋯O12^vi^	0.99	2.42	3.3910 (14)	167

Symmetry codes: (i) 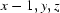; (ii) 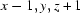; (iii) 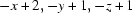; (iv) 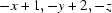; (v) 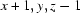; (vi) 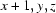.

**Fig. 1.**
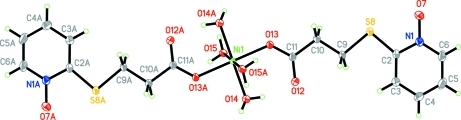
The molecular structure of the title compound, showing 50% probability displacement ellipsoids and the atom numbering scheme. Symmetry code: -x + 1,-y + 1,-z + 1.

**Fig. 2.**
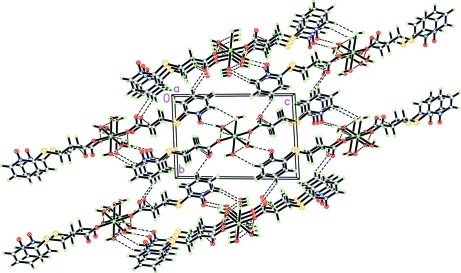
The crystal packing of the title compound, viewed down the a axis. Molecules are stacked along the a axis.

## supplementary crystallographic information

### Comment

N-oxides and their derivatives show a broad spectrum of biological activity such as antifungal, antimicrobial and antibacterial activities (Lobana & Bhatia, 1989; Symons & West, 1985). These compounds are also found to be involved in DNA strand scission under physiological conditions (Katsuyuki *et al.*, 1991; Bovin *et al.*, 1992). Pyridine N–oxides bearing a sulfur group in position two display significant antimicrobial activity (Leonard *et al.*, 1955). In view of the importance of N–oxides, we have previously reported the crystal structures of N–oxide derivatives (Jebas *et al.*, 2005; Ravindran *et al.*, 2008). As an extension of our work on N–oxide derivatives, we report here the crystal structure of the title compound.

The asymmetric unit  comprises of half molecule of the title compound, the other half is symmetry generated [symmetry code: -*x* + 1,-*y* + 1,-*z* + 1]. The Ni^II^ ion which lies on an inversion centre is six coordinated by four water molecules and two propianoto oxygen atoms from two 2-pyridylsulfanylpropionato N-oxide ligands forming a slightly distorted octahedral geometry. The bond lengths and angles agree well with the N–oxide derivatives reported earlier (Jebas *et al.*, 2005)

Intramolecular O—H···O hydrogen bonding influences the conformation of the molecule. The crystal packing (Fig. 2) is consolidated by intermolecular O—H···O and C—H···O hydrogen bonding together with intramolecular S···O = 2.6968 (10) Å; O···O = 2.6248 (12) Å, intermolecular O···O^i^ = 2.6469 (12) Å; O···O^ii^ = 2.6570 (12) Å and O···O^iii^ = 2.9455 (12) Å [symmetry code: (i):1 + *x*,*y*,-1 + *z*; (ii) 2 - *x*,1 - *y*,1 - *z*; (iii) 1 - *x*,1 - *y*,1 - *z*] short contacts. The molecules are stacked along the *a* axis.

### Experimental

A mixture of the potassium salt of 3(1-oxo-pyridinine- 2-sulfanyl)propionic acid (0.237 g,1 mmol) and Nickel (II) chloride (0.13 g, 0.5 mmol), in water (20 ml) was heated at 333k with continous stirring for one hour. The solution was kept aside for slow evaporation. After two weeks, green colored crystals were obtained.

### Refinement

After checking their presence in the Fourier map, all the hydrogen atoms were fixed on the calculated positions and allowed to ride on their parent atoms with the C—H = 0.95 Å (aromatic), C—H = 0.99 Å (methylene) and O—H = 0.81–0.82 Å (water) with *U*_iso_(C) in the range of 1.2U_eq_(C) and 1.5U_eq_(O).

### Crystal data

**Table:**

[Ni(C_8_H_8_NO_3_S)_2_(H_2_O)_4_]	*Z* = 1
*M**_r_* = 527.20	*F*(000) = 274
Triclinic, *P*1	*D*_x_ = 1.636 Mg m^−^^3^
Hall symbol: -P 1	Mo *K*α radiation, λ = 0.71069 Å
*a* = 4.8155 (5) Å	Cell parameters from 6946 reflections
*b* = 8.765 (1) Å	θ = 2.3–28.2°
*c* = 12.9560 (15) Å	µ = 1.16 mm^−^^1^
α = 86.400 (2)°	*T* = 173 K
β = 79.501 (2)°	Plate, green
γ = 84.929 (2)°	0.35 × 0.28 × 0.07 mm
*V* = 534.98 (10) Å^3^	

### Data collection

**Table:**

Bruker SMART APEXII CCD diffractometer	2615 independent reflections
Radiation source: sealed Tube	2501 reflections with *I* > 2σ(*I*)
graphite	*R*_int_ = 0.017
CCD scan	θ_max_ = 28.2°, θ_min_ = 1.6°
Absorption correction: multi-scan (*SADABS*; Sheldrick, 2008)	*h* = −6→6
*T*_min_ = 0.405, *T*_max_ = 0.492	*k* = −11→11
9627 measured reflections	*l* = −17→17

### Refinement

**Table:**

Refinement on *F*^2^	Primary atom site location: structure-invariant direct methods
Least-squares matrix: full	Secondary atom site location: difference Fourier map
*R*[*F*^2^ > 2σ(*F*^2^)] = 0.020	Hydrogen site location: inferred from neighbouring sites
*wR*(*F*^2^) = 0.055	H-atom parameters constrained
*S* = 1.05	*w* = 1/[σ^2^(*F*_o_^2^) + (0.0294*P*)^2^ + 0.2035*P*] where *P* = (*F*_o_^2^ + 2*F*_c_^2^)/3
2615 reflections	(Δ/σ)_max_ < 0.001
142 parameters	Δρ_max_ = 0.40 e Å^−^^3^
0 restraints	Δρ_min_ = −0.36 e Å^−^^3^

### Special details

**Table:**

Geometry. All e.s.d.'s (except the e.s.d. in the dihedral angle between two l.s. planes) are estimated using the full covariance matrix. The cell e.s.d.'s are taken into account individually in the estimation of e.s.d.'s in distances, angles and torsion angles; correlations between e.s.d.'s in cell parameters are only used when they are defined by crystal symmetry. An approximate (isotropic) treatment of cell e.s.d.'s is used for estimating e.s.d.'s involving l.s. planes.
Refinement. Refinement of *F*^2^ against ALL reflections. The weighted *R*-factor *wR* and goodness of fit *S* are based on *F*^2^, conventional *R*-factors *R* are based on *F*, with *F* set to zero for negative *F*^2^. The threshold expression of *F*^2^ > σ(*F*^2^) is used only for calculating *R*-factors(gt) *etc*. and is not relevant to the choice of reflections for refinement. *R*-factors based on *F*^2^ are statistically about twice as large as those based on *F*, and *R*- factors based on ALL data will be even larger.

### Fractional atomic coordinates and isotropic or equivalent isotropic displacement parameters (Å^2^)

**Table:**

	*x*	*y*	*z*	*U*_iso_*/*U*_eq_	
Ni1	0.5000	0.5000	0.5000	0.01405 (6)	
N1	1.2267 (2)	0.77325 (12)	−0.20598 (8)	0.0197 (2)	
C2	1.0696 (2)	0.79224 (13)	−0.10776 (8)	0.0167 (2)	
C3	0.8347 (3)	0.89789 (14)	−0.09608 (9)	0.0212 (2)	
H3	0.7229	0.9119	−0.0285	0.025*	
C4	0.7627 (3)	0.98279 (15)	−0.18222 (10)	0.0254 (3)	
H4	0.6027	1.0555	−0.1741	0.031*	
C5	0.9266 (3)	0.96061 (16)	−0.28069 (10)	0.0291 (3)	
H5	0.8794	1.0180	−0.3406	0.035*	
C6	1.1567 (3)	0.85564 (16)	−0.29110 (10)	0.0274 (3)	
H6	1.2685	0.8403	−0.3586	0.033*	
O7	1.44740 (19)	0.67209 (11)	−0.21521 (7)	0.0264 (2)	
S8	1.19805 (6)	0.67388 (3)	−0.01121 (2)	0.01821 (7)	
C9	0.9142 (2)	0.70873 (14)	0.09994 (8)	0.0176 (2)	
H9A	0.8880	0.8194	0.1136	0.021*	
H9B	0.7344	0.6765	0.0848	0.021*	
C10	0.9942 (2)	0.61698 (13)	0.19529 (8)	0.0177 (2)	
H10A	1.0268	0.5071	0.1796	0.021*	
H10B	1.1730	0.6511	0.2100	0.021*	
C11	0.7643 (2)	0.63642 (13)	0.29187 (8)	0.0159 (2)	
O12	0.55540 (18)	0.72858 (11)	0.28796 (7)	0.02341 (18)	
O13	0.80467 (17)	0.55410 (10)	0.37393 (6)	0.01937 (17)	
O14	0.26378 (17)	0.70791 (10)	0.47964 (6)	0.01973 (17)	
H14A	0.3264	0.7271	0.4179	0.030*	
H14B	0.0952	0.7007	0.4849	0.030*	
O15	0.71999 (16)	0.60521 (10)	0.59458 (6)	0.01836 (17)	
H15A	0.6207	0.6252	0.6514	0.028*	
H15B	0.8596	0.5522	0.6092	0.028*	

### Atomic displacement parameters (Å^2^)

**Table:**

	*U*^11^	*U*^22^	*U*^33^	*U*^12^	*U*^13^	*U*^23^
Ni1	0.01095 (10)	0.01935 (11)	0.01075 (10)	0.00213 (7)	−0.00158 (7)	0.00208 (7)
N1	0.0229 (5)	0.0205 (5)	0.0145 (4)	−0.0001 (4)	−0.0011 (4)	−0.0011 (4)
C2	0.0191 (5)	0.0181 (5)	0.0127 (5)	−0.0037 (4)	−0.0019 (4)	−0.0001 (4)
C3	0.0223 (5)	0.0220 (6)	0.0178 (5)	0.0001 (4)	−0.0012 (4)	0.0002 (4)
C4	0.0277 (6)	0.0222 (6)	0.0259 (6)	0.0029 (5)	−0.0068 (5)	0.0018 (5)
C5	0.0399 (7)	0.0273 (6)	0.0199 (6)	0.0009 (5)	−0.0090 (5)	0.0055 (5)
C6	0.0377 (7)	0.0291 (6)	0.0135 (5)	0.0006 (5)	−0.0017 (5)	0.0019 (5)
O7	0.0259 (4)	0.0308 (5)	0.0188 (4)	0.0075 (4)	0.0012 (3)	−0.0025 (3)
S8	0.01735 (13)	0.02289 (15)	0.01281 (13)	0.00103 (10)	−0.00070 (10)	0.00202 (10)
C9	0.0165 (5)	0.0228 (5)	0.0121 (5)	−0.0006 (4)	−0.0004 (4)	0.0017 (4)
C10	0.0159 (5)	0.0229 (5)	0.0131 (5)	−0.0003 (4)	−0.0016 (4)	0.0021 (4)
C11	0.0147 (5)	0.0205 (5)	0.0127 (5)	−0.0024 (4)	−0.0027 (4)	0.0011 (4)
O12	0.0214 (4)	0.0286 (5)	0.0168 (4)	0.0067 (3)	−0.0004 (3)	0.0053 (3)
O13	0.0137 (4)	0.0298 (4)	0.0129 (4)	0.0020 (3)	−0.0015 (3)	0.0052 (3)
O14	0.0164 (4)	0.0241 (4)	0.0169 (4)	0.0030 (3)	−0.0013 (3)	0.0019 (3)
O15	0.0147 (4)	0.0256 (4)	0.0139 (4)	0.0030 (3)	−0.0024 (3)	−0.0009 (3)

### Geometric parameters (Å, °)

**Table:**

Ni1—O13^i^	2.0488 (8)	C5—H5	0.9500
Ni1—O13	2.0488 (8)	C6—H6	0.9500
Ni1—O15^i^	2.0644 (8)	S8—C9	1.8165 (11)
Ni1—O15	2.0644 (8)	C9—C10	1.5216 (15)
Ni1—O14	2.0898 (8)	C9—H9A	0.9900
Ni1—O14^i^	2.0898 (8)	C9—H9B	0.9900
N1—O7	1.3154 (13)	C10—C11	1.5195 (15)
N1—C6	1.3579 (16)	C10—H10A	0.9900
N1—C2	1.3687 (14)	C10—H10B	0.9900
C2—C3	1.3889 (16)	C11—O12	1.2395 (14)
C2—S8	1.7405 (11)	C11—O13	1.2818 (13)
C3—C4	1.3823 (17)	O14—H14A	0.8142
C3—H3	0.9500	O14—H14B	0.8100
C4—C5	1.3877 (19)	O15—H15A	0.8216
C4—H4	0.9500	O15—H15B	0.8268
C5—C6	1.3685 (19)		
			
O13^i^—Ni1—O13	180.0	C6—C5—H5	120.2
O13^i^—Ni1—O15^i^	88.53 (3)	C4—C5—H5	120.2
O13—Ni1—O15^i^	91.47 (3)	N1—C6—C5	120.67 (11)
O13^i^—Ni1—O15	91.47 (3)	N1—C6—H6	119.7
O13—Ni1—O15	88.53 (3)	C5—C6—H6	119.7
O15^i^—Ni1—O15	180.0	C2—S8—C9	100.23 (5)
O13^i^—Ni1—O14	88.50 (3)	C10—C9—S8	108.10 (8)
O13—Ni1—O14	91.50 (3)	C10—C9—H9A	110.1
O15^i^—Ni1—O14	90.66 (3)	S8—C9—H9A	110.1
O15—Ni1—O14	89.34 (3)	C10—C9—H9B	110.1
O13^i^—Ni1—O14^i^	91.50 (3)	S8—C9—H9B	110.1
O13—Ni1—O14^i^	88.50 (3)	H9A—C9—H9B	108.4
O15^i^—Ni1—O14^i^	89.34 (3)	C11—C10—C9	111.71 (9)
O15—Ni1—O14^i^	90.66 (3)	C11—C10—H10A	109.3
O14—Ni1—O14^i^	180.0	C9—C10—H10A	109.3
O7—N1—C6	121.11 (10)	C11—C10—H10B	109.3
O7—N1—C2	117.75 (10)	C9—C10—H10B	109.3
C6—N1—C2	121.15 (10)	H10A—C10—H10B	107.9
N1—C2—C3	118.82 (10)	O12—C11—O13	124.27 (10)
N1—C2—S8	112.99 (8)	O12—C11—C10	119.85 (10)
C3—C2—S8	128.19 (9)	O13—C11—C10	115.87 (9)
C4—C3—C2	120.43 (11)	C11—O13—Ni1	126.08 (7)
C4—C3—H3	119.8	Ni1—O14—H14A	98.9
C2—C3—H3	119.8	Ni1—O14—H14B	114.4
C3—C4—C5	119.25 (12)	H14A—O14—H14B	107.5
C3—C4—H4	120.4	Ni1—O15—H15A	111.7
C5—C4—H4	120.4	Ni1—O15—H15B	113.8
C6—C5—C4	119.69 (12)	H15A—O15—H15B	105.3
			
O7—N1—C2—C3	−179.71 (10)	N1—C2—S8—C9	−171.71 (9)
C6—N1—C2—C3	0.08 (17)	C3—C2—S8—C9	7.83 (12)
O7—N1—C2—S8	−0.12 (14)	C2—S8—C9—C10	−178.14 (8)
C6—N1—C2—S8	179.66 (10)	S8—C9—C10—C11	−178.64 (8)
N1—C2—C3—C4	−0.39 (18)	C9—C10—C11—O12	−5.56 (15)
S8—C2—C3—C4	−179.90 (10)	C9—C10—C11—O13	175.00 (10)
C2—C3—C4—C5	0.4 (2)	O12—C11—O13—Ni1	20.56 (17)
C3—C4—C5—C6	−0.1 (2)	C10—C11—O13—Ni1	−160.03 (7)
O7—N1—C6—C5	179.99 (12)	O15—Ni1—O13—C11	−120.55 (9)
C2—N1—C6—C5	0.2 (2)	O14—Ni1—O13—C11	−31.25 (10)
C4—C5—C6—N1	−0.2 (2)		

Symmetry codes: (i) −*x*+1, −*y*+1, −*z*+1.

### Hydrogen-bond geometry (Å, °)

**Table:**

*D*—H···*A*	*D*—H	H···*A*	*D*···*A*	*D*—H···*A*
O14—H14A···O12	0.81	1.84	2.6248 (12)	162
O14—H14B···O15^ii^	0.81	2.27	2.9517 (12)	142
O14—H14B···O13^ii^	0.81	2.64	3.2316 (12)	131
O15—H15A···O7^iii^	0.82	1.83	2.6469 (12)	172
O15—H15B···O13^iv^	0.83	1.83	2.6570 (11)	172
C4—H4···O12^v^	0.95	2.48	3.2044 (17)	133
C6—H6···O14^vi^	0.95	2.46	3.2515 (16)	140
C10—H10B···O12^vii^	0.99	2.42	3.3910 (14)	167

Symmetry codes: (ii) *x*−1, *y*, *z*; (iii) *x*−1, *y*, *z*+1; (iv) −*x*+2, −*y*+1, −*z*+1; (v) −*x*+1, −*y*+2, −*z*; (vi) *x*+1, *y*, *z*−1; (vii) *x*+1, *y*, *z*.
